# Supplementary material for: Assessing the climate change exposure of foreign direct investment
Source: Nat Commun. 2022 Mar 18;13:1451. doi: 10.1038/s41467-022-28975-5 (PMC8933464; doi:10.1038/s41467-022-28975-5)
Supplement: Supplementary file 1 — Supplementary Information [file 41467_2022_28975_MOESM1_ESM.pdf]

Supplementary Information

Supplementary Fig. 1. Average Climate Risk Scores of Overseas Facilities by Host Country: Top 15 Countries by FDI Inflow Stock, 1970 - 2019

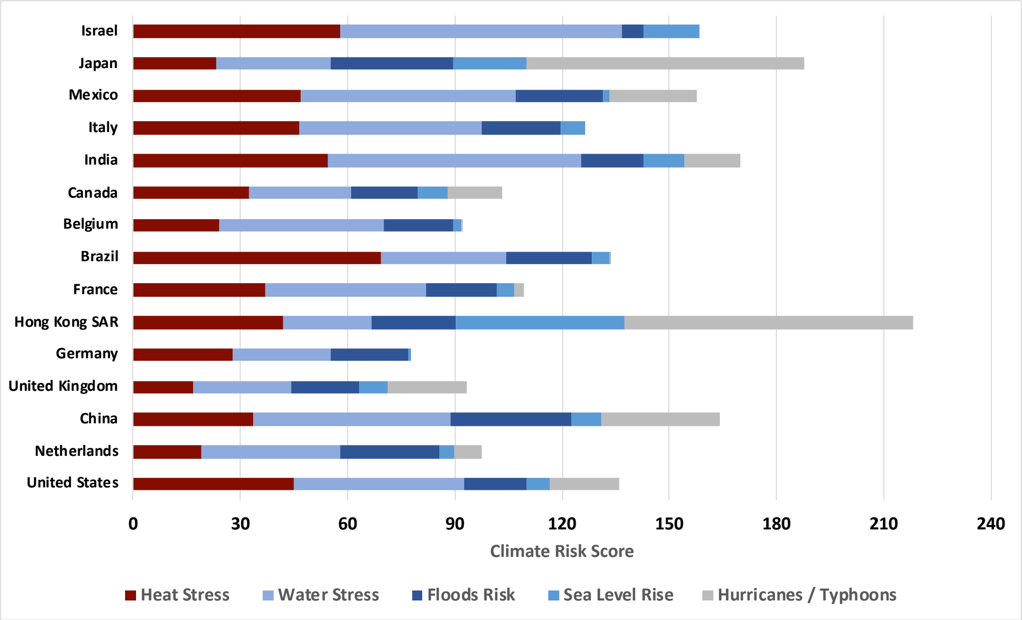

Analysis based on climate risk scores and facility statistics of 2,233 public companies from Four Twenty Seven. FDI inflow stocks are based on the World Bank data.

Supplementary Table 1. Climate Risk Drivers and 427 Methodology

Climate Risk Driver	Description	Spatial scale	Baseline period	Projection Period	Measurement
**Floods**	Change in rainfall conditions and size and frequency of possible floods	90 x 90 m (Flood) 25 x 25 km (Rainfall)	1975-2005 (Rainfall) 1985-2011 (Flood)	2030-2040 (Rainfall)	The flood score measures the severity and frequency of historical pluvial and fluvial floods, the frequency of future heavy rainfall events, and the intensity of prolonged periods of heavy rainfall. The flood frequency and severity indicators are based on a 1,000-year simulated history that was extrapolated from an observational history, and the rainfall indicators are based on global climate models.
**Heat Stress**	Increase in temperature	25 x 25 km	1975-2005	2030-2040	The heat stress score measures the relative change over time in the frequency and severity of hot days as well as in average temperature. Locations with high forecasted changes relative to recent history are most likely to be affected by temperatures unlike those previously experienced, even if these locations are not projected to experience the absolute warmest temperatures. Four Twenty Seven's analysis includes indicators related to changes in energy demand, changes in maximum temperature, and additional hot days.
**Hurricanes and Typhoons**	Exposure to past cyclones	25 x 25 km	1980-2016	N/A	The hurricanes & typhoons score captures geographic exposure to hurricanes or typhoons, also known as tropical cyclones. This cumulative measure reflects both the severity of storms with the highest maximum winds, but also the frequency with which an area has been subject to severe storms, excluding tornadoes and inland windstorms. Four Twenty Seven's uses a dataset of all recorded cyclones over the period 1980 and 2016. Only historical data is used, because global projection data is unavailable due to high uncertainty of how climate change influences tropical cyclone formation and intensity.
**Sea Level Rise**	Heightened storm surge, augmented by sea level rise	90 x 90 m	1986-2005	2040	The sea level rise score estimates the absolute and relative increase in the frequency of coastal floods. Estimates of sea level rise exposure are intended to capture: (1) the frequency of inundation due to a combination of sea level rise, storm surge, and high tides, and (2) change in the frequency of inundation between historical and projected periods. Estimates leverage global high resolution digital elevation model data as well as local storm surge and sea level rise estimates between 2017 and 2040. This analysis incorporates local flood risk statistics and local median sea level rise projections under carbon emissions scenario RCP 8.5.
**Water Stress**	Change in water supply and demand	Watershed	1950-2008	2040	The water stress score measures projected changes in drought-like patterns. It includes indicators that measure inter-annual variability, and absolute and relative percent changes in supply and demand for surface water (rainfall or rivers; not lakes or groundwater) available for consumptive use between the current period and 2040.

Supplementary Table 2. Descriptive Statistics

Variable	Obs	Mean	Std. Dev.	Min	Max
FacilityHeat	54,816	37.21	13.78	0.00	100.00
FacilityWater	54,364	40.61	13.05	0.00	100.00
FacilityFloods	53,864	22.37	10.54	0.00	100.00
FacilitySealevel	54,816	9.31	9.37	0.00	100.00
FacilityHurricanes	54,816	26.16	25.39	0.00	100.00
Cash	52,271	0.12	0.10	0.00	0.87
Size	52,804	10.36	1.51	4.03	15.25
ROA	51,651	0.11	0.07	-0.38	0.71
Leverage	52,767	0.25	0.17	0.00	3.89
GDPPerCapita	52,254	51.75	15.49	0.78	135.02
Co2PerCapita	53,081	9.74	4.60	0.04	38.61

Supplementary Table 3. Difference of Climate Risks of Chinese-owned/operated Overseas Facilities – Excluding Mining/Gas/Electric/Transportation Sectors

	Model 1 - Across Country					Model 2 - Within Country				
	Heat	Water	Floods	Sea Level Rise	Hurricanes/ Typhoons	Heat	Water	Floods	Sea Level Rise	Hurricanes/ Typhoons
ChineseFDI	0.00	0.06	0.28	0.08	0.50	0.03	-0.10	0.01	-0.06	-0.04
	[0.07]	[0.04]	[0.06]***	[0.10]	[0.14]***	[0.02]	[0.04]*	[0.01]	[0.09]	[0.07]
Controls										
GDPPerCapita	-0.07	-0.05	0.02	0.03	-0.03	0.00	0.01	0.01	0.03	0.00
	[0.03]*	[0.02]**	[0.01]	[0.01]*	[0.02]	[0.00]	[0.01]	[0.00]	[0.01]***	[0.01]
CO2PerCapita	-0.09	-0.06	0.00	-0.03	0.06	0.00	-0.02	-0.01	-0.05	0.00
	[0.02]***	[0.03]*	[0.01]	[0.01]*	[0.04]	[0.00]	[0.02]	[0.01]	[0.01]***	[0.01]
Cash	0.15	0.41	0.00	0.29	-0.66	-0.09	0.36	-0.09	0.33	-0.20
	[0.18]	[0.19]*	[0.06]	[0.16]	[0.16]***	[0.03]***	[0.14]**	[0.05]	[0.20]	[0.08]**
Size	0.00	0.02	0.02	0.05	0.03	0.00	0.01	0.02	0.06	0.00
	[0.03]	[0.02]	[0.01]*	[0.02]*	[0.03]	[0.00]	[0.00]	[0.01]*	[0.02]**	[0.00]
ROA	-0.04	-0.13	0.06	0.07	0.37	0.05	-0.02	0.01	0.1	0.12
	[0.65]	[0.37]	[0.04]	[0.40]	[0.17]*	[0.05]	[0.10]	[0.03]	[0.32]	[0.03]***
Leverage	-0.07	0.02	0.04	-0.01	-0.14	-0.02	0.07	0.07	0.01	-0.02
	[0.08]	[0.05]	[0.03]	[0.04]	[0.07]*	[0.01]	[0.02]***	[0.02]***	[0.03]	[0.02]
FirmLocalExp	0.03	-0.09	0.01	0.02	0.09	0.01	0.01	0.00	0.00	-0.01
	[0.01]*	[0.02]***	[0.00]***	[0.01]	[0.03]**	[0.00]	[0.01]	[0.01]	[0.01]	[0.01]
Host Country FE	N	N	N	N	N	Y	Y	Y	Y	Y
Industry FE	Y	Y	Y	Y	Y	Y	Y	Y	Y	Y
N	36470	36173	35427	36470	36470	36470	36173	35427	36470	36470
r2	0.07	0.14	0.12	0.05	0.04	0.95	0.77	0.36	0.46	0.91

The unit of analysis is firm-host country-industry. Standard errors are clustered at the industry level. Outcome variables are climate risk scores and are standardized to a mean of 0 and a standard deviation of 1.
***P<0.01, **P<0.05, *P<0.1

Supplementary Table 4. Difference of Climate Risks of Chinese-owned/operated Overseas Facilities – Different Sample: Top 15 Countries with Highest FDI Outflow Stock between 1970 and 2019

	Model 1 - Across Country					Model 2 - Within Country				
	Heat	Water	Floods	Sea Level Rise	Hurricanes/ Typhoons	Heat	Water	Floods	Sea Level Rise	Hurricanes/ Typhoons
ChineseFDI	0.02	0.09	0.26	-0.02	0.47	0.01	-0.08	-0.09	-0.18	-0.12
	[0.08]	[0.08]	[0.03]***	[0.11]	[0.09]***	[0.02]	[0.04]*	[0.06]	[0.11]	[0.08]
Controls										
GDPPerCapita	-0.21	-0.05	0.04	0.01	-0.01	-0.01	0.00	0.02	0.03	0.00
	[0.02]***	[0.02]**	[0.02]	[0.02]	[0.04]	[0.00]**	[0.01]	[0.01]*	[0.01]***	[0.01]
CO2PerCapita	-0.02	-0.07	-0.01	-0.03	0.04	0.00	-0.02	-0.02	-0.07	-0.01
	[0.03]	[0.04]*	[0.01]	[0.03]	[0.04]	[0.01]	[0.02]	[0.01]*	[0.02]***	[0.01]
Cash	-0.11	0.33	-0.05	0.31	-0.86	-0.08	0.38	-0.13	0.33	-0.21
	[0.20]	[0.23]	[0.06]	[0.16]*	[0.22]***	[0.03]**	[0.16]**	[0.05]**	[0.20]	[0.11]*
Size	0.00	0.01	0.02	0.06	0.03	0.00	-0.01	0.02	0.06	0.00
	[0.03]	[0.02]	[0.01]**	[0.02]**	[0.04]	[0.01]	[0.01]	[0.01]**	[0.02]***	[0.00]
ROA	-0.04	-0.19	0.08	0.32	0.69	-0.11	-0.29	-0.08	0.29	0.15
	[0.51]	[0.35]	[0.12]	[0.39]	[0.28]**	[0.18]	[0.21]	[0.11]	[0.31]	[0.04]***
Leverage	0.05	0.03	0.04	-0.04	-0.17	0.01	0.11	0.10	0.02	-0.02
	[0.06]	[0.04]	[0.03]	[0.05]	[0.04]***	[0.02]	[0.04]**	[0.02]***	[0.04]	[0.02]
FirmLocalExp	-0.10	-0.12	0.01	0.02	0.09	0.00	0.03	0.00	0.02	-0.01
	[0.01]***	[0.03]***	[0.01]*	[0.01]**	[0.04]**	[0.00]	[0.01]*	[0.01]	[0.01]**	[0.01]**
Host Country FE	N	N	N	N	N	Y	Y	Y	Y	Y
Industry FE	Y	Y	Y	Y	Y	Y	Y	Y	Y	Y
N	32306	31998	31672	32306	32306	32306	31998	31672	32306	32306
r2	0.09	0.14	0.12	0.05	0.05	0.94	0.76	0.36	0.45	0.90

The unit of analysis is firm-host country-industry. Standard errors are clustered at the industry level. Outcome variables are climate risk scores and are standardized to a mean of 0 and a standard deviation of 1.
***P<0.01, **P<0.05, *P<0.1

Supplementary Table 5. Difference of Climate Risks of Chinese-owned/operated Overseas Facilities – Different Control Variables

	Model 1 - Across Country					Model 2 - Within Country				
	Heat	Water	Floods	Sea Level Rise	Hurricanes/ Typhoons	Heat	Water	Floods	Sea Level Rise	Hurricanes/ Typhoons
ChineseFDI	-0.09	0.22	0.28	0.03	0.60	0.01	-0.01	-0.09	-0.13	-0.11
	[0.09]	[0.06]***	[0.04]***	[0.09]	[0.10]***	[0.02]	[0.03]	[0.07]	[0.11]	[0.06]*
Controls										
Cash	0.23	0.41	-0.01	0.32	-0.50	-0.07	0.32	-0.08	0.32	-0.19
	[0.21]	[0.21]*	[0.05]	[0.19]	[0.16]***	[0.03]*	[0.16]*	[0.05]	[0.21]	[0.08]**
Size	0.00	0.01	0.02	0.05	0.01	0.00	0.00	0.02	0.05	0.00
	[0.02]	[0.02]	[0.01]**	[0.02]**	[0.02]	[0.00]	[0.01]	[0.01]**	[0.02]**	[0.00]
ROA	-0.55	-0.61	0.16	-0.07	0.29	-0.10	-0.21	-0.09	0.03	0.09
	[0.60]	[0.38]	[0.08]*	[0.42]	[0.26]	[0.13]	[0.17]	[0.09]	[0.29]	[0.06]
Leverage	-0.02	0.01	0.01	0.00	-0.09	0.00	0.08	0.07	-0.01	-0.01
	[0.08]	[0.05]	[0.04]	[0.05]	[0.06]	[0.01]	[0.02]***	[0.02]***	[0.03]	[0.02]
Host Country FE	N	N	N	N	N	Y	Y	Y	Y	Y
Industry FE	Y	Y	Y	Y	Y	Y	Y	Y	Y	Y
N	42911	42506	42067	42911	42911	42911	42506	42067	42911	42911
r2	0.06	0.11	0.12	0.04	0.03	0.94	0.75	0.36	0.44	0.89

The unit of analysis is firm-host country-industry. Standard errors are clustered at the industry level. Outcome variables are climate risk scores and are standardized to a mean of 0 and a standard deviation of 1.
***P<0.01, **P<0.05, *P<0.1

Supplementary Table 6. Difference of Climate Risks of Chinese-owned/operated Overseas Facilities –Combined Climate Risk

	Across Host Country	Within Host Country
	**Total Climate Risk**	**Total Climate Risk**
ChineseFDI	0.22	-0.28
	[0.11]*	[0.11]**
Controls		
GDPPerCapita	-0.05	-0.01
	[0.02]***	[0.00]
CO2PerCapita	-0.02	0.00
	[0.01]	[0.01]
Cash	0.28	0.15
	[0.10]**	[0.08]*
Size	0.01	0.00
	[0.02]	[0.01]
ROA	-0.19	-0.23
	[0.35]	[0.26]
Leverage	-0.03	0.08
	[0.05]	[0.02]***
FirmLocalExp	0.03	-0.01
	[0.01]*	[0.01]
Host Country FE	N	Y
Industry FE	Y	Y
N	39495	39495
r2	0.15	0.80

The unit of analysis is firm-host country-industry. Standard errors are clustered at the industry level. Outcome variables are climate risk scores and are standardized to a mean of 0 and a standard deviation of 1.
***P<0.01, **P<0.05, *P<0.1

Climate Risk Adjustment Based on Facilities' Economic Activities

Four Twenty Seven applies two adjustments on facilities' climate risk scores: one for heat stress and another for water stress. These adjustments are made based on research about the impacts of heat stress on labor as well as a Multi-Regional Input-Output (MRIO) model[1] that informs whether certain facilities in one country are resource or water intensive than those in other countries. Water stress adjustments are based on the water demand requirements according to specific combinations of the asset's sector and country of operation. These estimates are derived from a MRIO model. Heat stress adjustments account for the energy demand requirements across different sectors according to MRIO and consider whether a sector is likely to depend upon outdoor or labor-intensive activities, which are sensitive to extreme heat conditions. The scale of the adjustments ranges from 0.75 to 1.25, providing an equal discount ($x<1$) and penalty ($x>1$) of 0.25 to those facilities that fall above or below average ($x=1$) for all sectors.[2]

References

1. Lenzen M., Moran D., Kanemoto K. & Geschke A. Building Eora: A Global Multi-regional Input-Output Database at High Country and Sector Resolution. *Economic Systems Research* **25**(1): 20-49 (2013).

2. Graff Zivin J. & Neidell M. Temperature and the allocation of time: Implications for climate change. *Journal of Labor Economics* **32**(1): 1-26 (2014).
